# The College Bill Fully Discussed

**Published:** 1919-02-01

**Authors:** 


### NURSES' FIGHT FOR STATE REGISTRATION.

### THE COLLEGE BILL FULLY DISCUSSED.

### Representative Meeting in London: Plea for Unity.

An open meeting of nurses, convened by the College of Nursing, Limited, was held at the Home of the Royal Society of Medicine, on Thursday, January 23, when some 500 ladies attended, the largest hall being crowded.

The chair was occupied by **Sir Arthur Stanley,** G.B.E., C.B., who, in opening the proceedings, said :

Ladies and Gentlemen : My own task is a very light and short one, because I really have only to introduce the speakers. Still, I would like to express one word of welcome to those who are here to-night. First of all, I am delighted to see such an excellent gathering : I should think it is not far wrong to say this is the best and most representative gathering of nurses that has ever been held. (Applause.) We are here to discuss a matter of infinite importance to the nurses, and I am very glad that so many of them have attended to assist us in our deliberations. I want to say just one other word of welcome, and that is to those members of the College—and possibly, I hope, others—who have visited for the first time our new quarters at 7 Henrietta Street. It is the first real home of the College of Nursing. I have assisted at the beginning of a good many similar undertakings, such as the Red Cross, the Royal Automobile Club, and others, and we have grown into very big premises, but I find we always look back—that is to say, those who were founders look back—with pride and affection upon the first home, the first centre of our affections. And I feel perfectly certain that though—wisely as I think—we have begun in a small way at No. 7 Henrietta Street, eventually we shall have much bigger and finer premises, but we shall always keep warm hearts for our times at 7 Henrietta Street.

Nearly 12,000 Members.

As to the subject we have met to discuss, I have only one word to say. We, of the College of Nursing, set to work to try and get Parliament to pass a Registration Bill for Nurses, because we were convinced it was badly wanted. We did not differ in fundamentals, in aims and objects, from those who have been trying for many years to get a State Registration Bill, but we did differ to a certain extent in methods of procedure. We thought it was advisable to set to work to make a Register ourselves, to thrash out all the difficult questions which undoubtedly existed in connection with the formation of a Register : that when we had thrashed out those questions and prepared our own Register, then to go to Parliament to show that a Register of Trained Nurses—three-year certificated nurses—could be formed on lines acceptable to the nurses themselves, then ask Parliament to give statutory power to a Register so formed. We have done what we set out to do. To-day on our Register of the College of Nursing there are very nearly 12,000 members. (Applause.) Of those 12,000, or nearly 12,000, all except a very small minority—something like ten laymen and a few doctors—are properly registered certificated nurses of three years' training. (Applause.) Now, if we, as a voluntary society, can do that in two years, surely there ought to be no difficulty in persuading the State that it is possible to form a State Register of Nurses, and one which would be acceptable to the nurses themselves.

A Carefully Drawn Bill.

On those lines we have prepared the machinery. We have drawn a Bill, and that is the Bill you are met to-night to discuss. This Bill has been the subject of the most careful consideration by our Council, and by our Registration Committee, and all our members who were interested in the work of State Registration. I will ask one person who, possibly, knows it better than does anybody else, to briefly give you an outline of the Bill. I refer, of course, to Sir Cooper Perry, without whose help it would have been very difficult to arrive at the stage at which we have arrived at the College of Nursing. After he has spoken about the Bill he will be followed by Professor Glaister, who has been kind enough to come all the way from Scotland to give us the benefit of his assistance and advice. Then we shall have remarks by Miss Musson, who is well known, I think, to all of us. After that we invite questions and criticisms. We only want to place before you the Bill as it stands, to explain the reason, the why and the wherefore, of the different clauses in the Bill, and to invite questions and criticisms. I hope that at the end of the evening you will agree we have done our best to answer those questions fully and fairly. I call upon Sir Cooper Perry.

#### The Bill Explained.

**Sir Cooper Perry,** M.D. : Ladies and Gentlemen, our Chairman has been good enough to ask me to open this discussion by explaining, in such detail as time allows, the Bill which the College of Nursing is introducing for the training and registration of nurses, the Bill promoted by the College of Nursing, Limited, a Company limited by guarantee, as it is not perhaps superfluous to remark, having no share capital, and prohibited by its Memorandum of Association from making profits or paying anything by way of bonus or dividend to its members. The Bill for the Registration of Nurses is an integral part of the College programme, and in explaining it I must take you for a moment back to the circumstances which led to the initiation of the movement, in which our Chairman took the leading part, for endeavouring to establish what, if success crown our efforts, will become—I think I may almost venture to say has become—the central organisation of the nursing profession in all its branches throughout the United Kingdom. The project of the College was launched by Sir Arthur Stanley in the last days of 1915 when, in a circular letter to the various training-schools and the leading hospitals throughout the kingdom, he invited them to come to his assistance, having, as he wrote, become struck by the total lack of organisation among the various authorities responsible for the training of nurses, and by the need of co-ordinated effort amongst the nurses themselves, adding that in spite of more than twenty years of more or less active agitation in favour of registration of nurses, the movement, through causes which he deemed it inexpedient to enter, had hitherto failed to attain the objects sought.

Self-Government the Aim.

This original movement for the registration of nurses, in which many of those present at this meeting have taken an honourable part, was based upon an attempt to organise and marshal the scattered units of nurses throughout the country, and to provide them, through the Central Committee for the State Registration of Nurses, with a common meeting ground whence, by active propaganda, Parliament might be induced to pass a Nurses' Registration Bill.

**Nurses' Fight for State Registration**—(*continued*).

On the other hand, the College movement was initiated as a scheme of co-operation amongst the nurses' training-schools throughout the country. It was to be a purely voluntary body which would aim at securing the support and sympathy of the governors of hospitals to which nurse-training schools were attached, the matrons and teachers of those schools, the leading members of the medical profession, and last, not least, the trained nurses themselves. In accordance with this programme, the first Council of the College was formed, largely from amongst the matrons of the leading training-schools, the leading hospitals and infirmaries, both in London and in the country, in Scotland and in Ireland. Primarily, the scheme was based upon the co-operation of the matrons and lady superintendents of the leading nursing schools, whose knowledge and experience in matters of curriculum and examination were made use of to initiate the undertaking. It was recognised from the first that the College was ultimately to be self-governed. Already the original Council has become directly elected by the nurses on the Register as to one-third of its numbers, and by next year, when it can be said to have attained maturity, it will have become broad-based and entirely democratic in constitution. As will be seen from this sketch, the College is primarily an educational body, but with a very large and far-reaching programme for the professional advancement and social betterment of the nursing profession. Such a programme is far too big to be compressed within the clauses of a Registration Bill, and consequently it is essential, from the College point of view, that the General Nursing Council should not be limited to a mere matter of training and registering nurses, or protecting a title, a badge, and a uniform, but should be enabled to pursue all those other activities included in its Memorandum of Association, under which I may mention the provision of scholarships, post-graduate lectures, studentships, and those schemes for the benefit of nurses incapacitated by sickness, or who require assistance in old age, and who, during their active years, are constantly feeling the want of some organisation which will bring legitimate pressure to bear wherever nurses are employed, to secure for them better salaries, longer holidays, improved status, and at the same time will make it possible by registration for the community to have an assurance that the nurses they employ are skilful in their work, and have attained a certain level of professional competence.

LEADING TRAINING-SCHOOLS' SUPPORT.

The passing of a Registration Bill is but one item, though a most important item, in the programme of the College. I would remind you that at the present time the nurses of the best London and provincial hospitals and infirmaries are able to make a living on the reputation of the nursing certificates they hold from their own schools, and thus they have at present comparatively little inducement to enter for—and incidentally to pay fees for—any further qualifying certificate, or for registration itself. Hence the importance to the scheme of the countenance and active participation of the authorities of the leading nurse-training schools, for without their support and influence the nurses of the best schools will be only too apt to neglect registration altogether, or to regard the presence of their names on a common register as a mark rather of inferiority than distinction: and it is almost pathetic to contemplate the slow progress which the movement made whilst unconnected with nursing schools, with the rapid acceleration in interest and membership which has been manifested since the inception of the College on lines, as I have said, drawn so as to enlist the sympathy of the nurses' training-schools, to attract their nurses, and, having secured a representative body, to entrust future developments to a Council directly elected by nurses on the register.

STANDING FAST BY PLEDGES.

Such being a short history of the origin and course of the movement with which the College is associated, the lines upon which the College Bill is drawn become more recognisable. It is obviously necessary to keep in existence the College, with all its activities. It is equally necessary that in any Bill which the College promotes, or with which it associates itself, nurses who are on the Register of the College and whose qualifications have been verified by careful examination, should be included, without further fee, upon the first legal Register. I say "included upon the first legal Register." They claim no privileged position. Every other nurse, and on easier terms, for many years will be able to find her place on the legal Register. It is necessary, further, that a majority of the General Nursing Council should be elected directly by them. These are the pledges the Council has given to its members, and by them we stand fast, and on these principles there can be no compromise. But if the legal Register is to be kept, as we contend it should be kept, by the College of Nursing, the College of Nursing must consent to accept the Council appointed by the State, a Council elected not only by nurses, but by those other public bodies and authorities which may properly claim to be represented. That is one part of the price which the College pays for being permitted to keep the legal Register. The other part is to hand over all its funds, with property of every sort, to the General Nursing Council: in other words, to merge its own private interests in the interests of the nursing profession at large, whether members of the College or not.

OPPONENTS OF STATE REGISTRATION.

And now I turn to another consideration, which has a most important bearing on the structure of our Bill, and as to which we are almost equally at issue with the Central Committee. In this assembly we all of us are satisfied that the legal registration of nurses is an object for the attainment of which years of struggle and sacrifice are not too heavy a price. We are apt to forget that there are many outside these walls not, perhaps, saying much just now, indeed, lying low, who are by no means convinced that State Registration is a good thing. We have honourable opponents who object, on principle, to State interference, regarding the influence of the State as cramping private initiative, as a stereotyped, rigid uniformity of system, and generally tending to that regimentation of all human activities of which bureaucratic Germany was the greatest example. Nor has their objection to State Registration diminished by the experiences we have gone through at the hands of various Controllers who have lately been placed over us, perhaps for our good, but certainly at times for our chastisement. With these opponents we must be content honourably to differ.

But there are others whose opposition is more insidious. There are, throughout the country, managers of various hospitals—more than we know—who see in State Registration an increase of expenses in the way of equipment, in the way of shorter hours of duty to enable nurses to go in for examinations, a larger staff and larger salaries for teachers, and a curriculum less fashioned by their own mind. There are those who see in State Registration a drying-up of the sources whence they derive their probationers. Special hospitals, particularly the children's hospitals, are very anxious on this point. They see that

**Nurses' Fight for State Registration—**

if probationers are not to be obtained cheaply it will be necessary to employ, at larger salaries, fully-trained nurses helped by wardmaids. And, finally, there are those who fear that State Registration, by insisting on a three years' curriculum, may make it difficult to justify the sending out of probationers to do private nursing during their training, on the plea that two years at a particular hospital teaches them as much as three years elsewhere. (Laughter.) These opponents of State Registration are, as I have said, a more insidious danger to us, because they will masquerade, in Parliament and elsewhere, as our friends. They will be anxious to improve a State Registration Bill, and will be so full of suggestions that—no doubt to their infinite disappointment—the Bill will be talked out. And if we are to avoid this very real danger, our Bill should be, and indeed has been, submitted from time to time to those best able to advise us as to Parliamentary procedure. It has been moulded by their counsel throughout, and even now, perhaps, has not attained its final form, though the draft before you is the seventh—and seven, as all theologians know, is a perfect number. What, however, these authorities have, from the first, been unanimous in urging, is that in the present congested condition of Parliamentary business, if the Bill is to have any chance of passing it must be as short as possible: it must confine itself to essentials, and it must avoid unnecessary details.

The Central Committee's Bill Criticised.

That is what the College Bill seeks to do, and what a study of the Central Committee's Bill shows it does not do. I may say, if it is true that, as a Greek wrote, a large book is a large evil, the book of the Central Committee Bill is twice as large an evil as our College Bill. And now, having said so much, I will just illustrate my point by reference to only one matter in the Central Committee's Bill—the structure of the General Nursing Council. Now, in our own Bill, the structure of the General Nursing Council is left so far indeterminate that we merely set out certain authorities who must be represented, without giving any detail as to the proportion in which they shall be represented, and saying no more with regard to the General Nursing Council Constitution than that two-thirds of it shall be elected by the nurses, and we give the proportions in which they shall be elected from England and Wales, Scotland and Ireland. I would call your attention, by way of contrast, to the infinite detail which is elaborated in the Central Committee's Bill. The Council is of forty-one: they are elected or appointed as follows—the clauses run from "a" to "k." Those clauses may, every one of them, invite debate, but I will only mention to you one or two which have attracted my own attention. I notice that there is to be one medical practitioner elected by the medical superintendents of the fever hospitals. May it not reasonably be asked "Why should not the medical superintendents of the lunatic asylums elect a representative?" I do not say it could be reasonably asked, but it is a thing upon which, obviously, debate might be challenged, which might lead to a long discussion. Now take another point. There are four persons to be elected by the nurse-training schools—four out of forty-one. Of those four persons two shall be elected by the whole of the training-schools in England. Ireland is to have just half the representation of England, in other words, one. Again, there are to be eighteen registered nurses appointed by the nurses on the Register, eighteen out of forty-one—a very large proportion compared with the two-thirds which our Bill contemplates! But note: of the eighteen, England takes eight, Ireland, with a total population less than London, elects four. I am not arguing at this moment that that is inequitable, but what I do say with complete confidence is, that any such clause in a Bill will produce so much discussion, it may generate so much heat and friction—Irish matters often do—(laughter)—that little progress will be made, and there is little chance that the Bill will be passed. And there is another thing which interests and amuses me, namely, that those who are promoting the Bill of the Central Committee have, as many others have, great sympathy with democratic aspirations and with the Labour party, and yet are most anxious to prevent the nurses using their own judgment and electing the people that they want to elect! ("Shame.") They lay down that of those eight elected members for England, four of them shall be matrons. I am not saying anything against matrons, far from it. Do I not live with a matron? But I know this, that it has been a reproach to the College of Nursing that it has so many matrons upon its Council. Now we are going to see in this Bill of the Central Committee, which should be, and I have no doubt is intended to be, democratic, that of eight representatives, four are matrons and four are nurses.

At this point, Sir, I close, for I see that, though I might say more, I have exceeded the time, and I give way to Professor Glaister who, no doubt, will pursue a leisurely dissection of the Bill of the Central Committee. (Applause.)

### Have Done With Talking.

**Professor Glaister** (Glasgow): Mr. Chairman, Ladies and Gentlemen. I do not propose to follow Sir Cooper Perry in what he has said, because it would be a mere multiplication of words. He has expounded his part of the subject so lucidly that it is entirely unnecessary for me to repeat a single word, or go over any point which he has tackled. Therefore I propose to look at the Bill now before the public in one or two other aspects, and from one or two different points of view.

I suppose I am right in declaring that most of us here are agreed that the time is more than mature for a State Registration of Nurses. I apprehend that your attendance to-night is an evidence that you are all keen on seeing that nurses should be registered by the State, in order that you should have a proper footing in your profession; and, secondly, in order that the public may be able to discriminate between the woman who is a fully trained and certificated person and the woman who is not, but who puts up her plate indicating that she is. If that be true, then it seems to me that this Nurses' Registration movement has gone through a great many years of unnecessary talking. We are all out for State Registration, whether in our Bill or in any other Bill, and the question comes to be now, cannot we focus the whole matter and get on with the Bill? (Applause.) I am very keen to see that we should accomplish more than speaking and writing about it. A great deal of ink has been spilled, and a great deal of language has been spoken about this State Registration, and in this year of grace 1919 we are about as near as we were fifteen or more years ago when I first started to take an interest in the subject. That is not as it should be. I do not think the people who have had charge of the movement have been altogether to blame. I am bound to say frankly, in a meeting of nurses, that a good deal of the blame is attachable to themselves. (Hear, hear.) They have been apathetic, they have been lethargic, they have left others to do the delving and the ploughing, and they themselves have waited to reap. I want them to do some of the delving and the ploughing,

**Nurses' Fight for State Registration**—(*continued*).

I want them to indicate to Parliament by resolutions, by petitions, and by representations that State Registration is more than due, and that Parliament owes it to them to have it. Until nurses do that throughout the country and show some reason for the faith that is in them, you will hardly blame Parliament for lying low and saying, "Look here, come to us with an agreed Bill and we will pass it." Did you ever see, except during war-time, any measure in Parliament which was an agreed Bill? I never did that I recollect. It was only during the war that people became agreed to Bills. Bills are things which are made to evoke differences of opinion—(laughter)—and it is only when they come into the Houses of Parliament that people thrash differences out, and when they come to what is passed as an Act, namely an agreed document, it becomes an enactment of the State.

Scotland Tired of Waiting.

There are one or two points which I want to allude to for a moment. I should not like to say a single unkind word about any movement which has been parallel to that of the College, because I have been associated with another movement for a very long time. I have felt that any movement which helped towards State Registration deserved the assistance of myself or any one else who was interested in the subject of State Registration. But as you know, a long lapse of time took place after the meeting with the then Leader of the House of Commons, who told us to come with an agreed Bill because Parliament had no time for any other kind of Bill. It was then after long inaction that the movement began to which Sir Cooper Perry referred, and which he has now explained to you. In Scotland we were anxious to have State Registration, and the Association to which I have the honour to belong maintained its connection both with the other Committee and with the College, in the hope that an agreed Bill would be the result. Unfortunately, however, such an agreement has not been arrived at. And just the other day, tired of waiting for this *rapprochement*, our Association in Scotland unanimously resolved to dissociate itself from the other body and to ally itself entirely to promote the movement of the College of Nursing. (Applause.)

Provisional Councils Compared.

I do not want to say anything about the Provisional Council, or any other body, except this: The College of Nursing has tried to meet our friends, and I want to show how fairly we have tried to do it. When the Provisional Council has to be appointed under each Bill the membership of it in the College Bill is as follows: Three by the Council of the British Medical Association, three by the Council of the Medico-Psychological Association, three by the Council of the Association of Poor-Law Unions, three by the Council of the British Hospitals Association, twelve by the Central Committee for the State Registration of Nurses, and twelve by the Council of the College of Nursing, Limited. What do I find in the position of the other Provisional Council which is to be formed of twenty-eight? Three by the Council of the British Medical Association, one by the Medico-Psychological Association, none by the Council of the Association of Poor-Law Unions, none by the Council of the British Hospitals Association, ten persons by the Central Committee, two by the College of Nursing—(laughter)—three by the Privy Council, three by the Local Government Boards of England, Scotland, and Ireland respectively, one by the Admiralty, one by the War Office, one by the Queen Victoria Institute for Nurses, one by the Asylum Workers' Association, two by the Royal British Nurses' Association. It does not require any consideration at all to see the relative fairness of these proposals. You cannot very well give a man more than half of what you have, and you cannot do better for him than give him half an interest in any movement. But when it comes to ten to two, the arithmetical proportion is vastly different. There is another point regarding this Council. This Provisional Council in the College Bill has to do a certain definite little bit of work. It has got to regulate its own proceedings, and by that I mean arrange its rules for this, and after it has got that it has got to go to the electorate, namely, the nurses, to have the General Nursing Council passed. In the other Bill the Provisional Council has to deal with the whole of the matter mentioned in the Bill from nearly A to Z, or to be more exact, from E to M. They expect to take two years to do that, and at the end of two years the General Nursing Council comes to be elected. I am not sure that we are all willing to wait for two years after any Bill is passed before we get on with the business of registration.

Supplementary Registers.

Another point which is forced upon me with regard to this Bill is: I have heard, and probably most of you have heard, that there is an objection taken to the College Bill in that it takes powers to form supplementary registers. Now let us understand this matter perfectly clearly. When one goes to Parliament for a Bill the lawyers take very good care to put into a Bill a good deal more than they immediately want, at least in the way of asking for powers, because naturally man is supposed to be progressive, and if in the future something should emerge then unknown to them by which a Supplementary Register may have to be formed, you then have power in your Bill to do it. But you cannot have power until you have gone to the Privy Council and assured them that it was right, and so get their approval. In the other Bill there is no power to take a Supplementary Register. In Clause 13 of the Central Committee's Bill this is the language employed: "Any person who claims to be registered under this Act shall be entitled to be so registered, provided that such person is a British subject, at least twenty-one years of age, of good character, and"—listen to this—"has had not less than three years' training under a definite curriculum prescribed by the Council in the wards of a hospital or hospitals approved by the Council or any institution." If you turn to Clause 11 of the Bill and look at the letter (f), you will find that one of the duties of the Council is as follows: "Prescribing the conditions necessary to be fulfilled by any hospital desirous of having any portion of its training recognised *pro tanto* towards the three years' training required under the Act." That is the language of the Central Committee Bill. It is possible that they may believe that under these powers, if they think a Supplementary Register is needed in the future, that these words cover the possibility of making a Supplementary Register without putting the words into the Act. In the College Bill the words are these—they are both the same up to this: "The applicant must have had not less than three years' general training under a definite curriculum"—general training—"in a nurses' training-school or schools recognised by the General Nursing Council, and passed the examinations prescribed by the General Nursing Council." These words do not look, at first sight, very different, but they may portend much more than one sees at the present moment.

The Case of the V.A.D.s.

I do not want to say very much more, but I would like to add this. I have heard, and I daresay others have

**Nurses' Fight for State Registration—(continued).**

heard, a recent statement going around to the effect that the College of Nursing is out to confer some special favour on the ladies who are commonly spoken of as "V.A.D.s." May I say, in the presence of the Chairman of the Council, of the Council and with their authority, that it is not the intention of the College of Nursing to give any favour to any V.A.D. nurse until she has fulfilled the conditions required in that Bill. (Applause.) Please understand this. This Council of the College as well as the public, and I am sure every one of you here, give every honour and praise to those ladies who came forward to help the State and did such excellent work during the time the war was on. (Applause.) But we must be just before we can be generous, and I want to make myself perfectly clear on that point.

There are other points which may emerge after we have heard what my neighbour has to say about it, and therefore I content myself now with simply covering these two or three points and asking the ladies present, whatever views they may have with regard to this Bill or the other Bill, to do all they possibly can to press forward this question of registration upon the State, and try to let us have the measure in reasonable time. Some of us are growing old in this business, and we do not want to die before we see it achieved. (Applause.)

### A Matron's Point of View.

**Miss Musson :** My remarks will be as brief as our Chairman could possibly desire. He ensured that by putting me up to speak after Sir Cooper Perry and Professor Glaister. They have covered the ground so fully that nothing really remains for me to say. Nevertheless, I shall repeat things which they have said, and I make no apology for doing that because I look at things from a rather different point of view, and I may by chance put matters in a way which my fellow-nurses will understand more readily than when put by others.

You know that the State Registration of trained nurses has been for many years a subject of the greatest interest to me. I consider it of such vital importance, not only to the nursing profession but also to the general public, that I would do anything I possibly could to get this put into a Bill. My chief reason for joining the College of Nursing, Limited, was that it appeared to me likely to hasten this reform. After many years of somewhat disheartening work the Bill brought forward by the Central Committee seemed no nearer becoming an Act. A suggestion which was made by our present Chairman appealed to me as being full of common sense. It was that instead of waiting for Parliament to give us authority to make a Register we should begin to make a Register on definite lines, and in that way prepare an electorate for the time when a Bill might be passed. This the College proceeded to do, and I hope the College is training the nurses to exercise the right of voting which the College gives to each individual member. We have now a membership of nearly 12,000, and I want to impress on everybody that the great majority of these members are nurses holding a three years' certificate from a recognised training-school, either general or Poor-Law, together with a few others whose training and experience have been accepted under the regulations applicable during the period of grace only. You all know that we have always recognised the fact that a certain number of nurses have been trained under old conditions; you know that the three years' standard is comparatively recent. There are nurses who were trained thirty years ago when only a one-year certificate was given, and it would be unfair to debar them because they trained before the three years' standard was accepted. Therefore after due inquiry these nurses are included in the Register as they would have to be included in any Register which was drawn up under an Act. These nurses have their certificates and references most carefully investigated before their names are submitted to the Council of the College for election. This is a very big piece of work, and has entailed very hard work; it has kept, and is keeping, several people going. And I think it will be readily understood that all this is so much to the good. We are getting on with the work; it would have to be done sooner or later.

### Democratic Election Methods.

In addition to the Register, the College has drafted this Bill for the State Registration of Trained Nurses, in support of which I am trying to speak from a nurse's point of view entirely. In a profession such as ours, in which the members are not only scattered but, by reason of their occupation, are frequently unable to attend meetings, the only democratic method of electing representatives is by postal ballot. This is the method adopted by the College of Nursing, like all other societies which begin with a nominated Council. One-third of the Council has already retired, and that one-third of the members has been replaced by others by the postal ballot. Very shortly another one-third will retire, and by next year there will be no one on the Council except those elected by the postal ballot—always excepting anything unforeseen which may occur by retirement or otherwise, and then the Council may nominate some one to take their place. No other candidates are nominated by the Council. The members of the Council are nominated as well as elected by the members themselves. All members of the College have equal right; matrons have no power greater than that of the latest qualified nurses. I say this not without thinking—I say it deliberately—that this is the most democratic body of nurses with which I have been connected. (Applause.) You would not expect me to, and I do not intend to, say one word—I would not for anything—against the societies which are known as self-governing societies supporting the Central Committee's Bill. I have belonged to three of them. Though, to my sincere regret, I am at present at variance with them, it must be conceded that all the attempts at organising the nursing profession in this country before 1915 were made by those societies, and to them belongs the honour of having broken up the rough ground. (Applause.)

### Public Challenge Investigated.

Statements, however, have been made which lead to great complications; a public challenge had been made that these societies claim to be more representative of nurses than the College of Nursing. I think it is, therefore, as well to investigate that claim. These societies are : the Royal British Nurses' Association, the Matrons' Council of Great Britain and Ireland, the Society for State Registration of Nurses, the National Union of Trained Nurses, the Irish Nurses' Association, the Scottish Nurses' Association, and the Fever Nurses' Association. The first of those, I think, does elect its own governing body by ballot. I think it has a membership of 3,000 to 4,000. The Matrons' Council is not representative of the rank and file of nurses; it is composed of present matrons and superintendents, and the members are proposed and seconded and elected by show of hands at the quarterly meeting. The President, the Hon. Treasurer, and the Hon. Secretary have power to deal with such business as may arise between quarterly meetings. It is a society which gives opportunity for pleasant and helpful meetings

**Nurses' Fight for State Registration**—(*continued*)
between matrons, but they are not sent there by the nurses, and therefore it is not a representative nurses' society. The Society for State Registration consists of a number of nurses who have produced evidence of training, and pay a subscription of 1s. a year, and of other persons who are interested in registration. The Executive Committee is not elected by ballot, but by a show of hands at the general meeting, and is, in fact, nominated by itself—which is practically inevitable in so scattered a profession as ours. In the case of the National Union of Trained Nurses, the members elect their own representatives by show of hands at meetings, not by ballot. The Irish Nurses' Association and the Scottish Nurses' Association I am not familiar with, but I think that neither of them has a postal ballot for the election of Council. The Fever Nurses' Association represents nurses with special training only, and is not representative of the general rank and file of the nursing profession. I therefore think that the College of Nursing may claim to be as independent and as self-governing as the societies composing the Central Committee. Further, in the College of Nursing, Limited, no person can be represented twice over, whereas on the Central Committee one person may be represented three or four times; she may be, and often is, at the same time a member of the Matrons' Council, of the State Registration Society, and of the Royal British Nursing Association, or of one or other of those bodies.

The Two Bills Compared.

In a leaflet in my nands it is stated that in the Bill promoted by the College it is proposed to incorporate the policy of control by governors of hospitals, matrons of hospitals, Poor-Law Guardians, and other officials. It also states that number one is the Workers' Bill and number two is the Employers' Bill. Well, I always thought I was a worker, and I am certainly not an employer. (Laughter.) I have been rather looking into the point of view of this second one being an Employers' Bill and the other the Workers' Bill. The Bill drafted by the College of Nursing gives one-third of the total number to representatives of public bodies and training-schools, one-third to the Central Committee, and one-third to the College. This gives for the Central Committee twelve representatives, which is the same number as claimed for them in their own Bill. For the constituent societies, such as the Royal British Nursing Association, they are not so generous to us. They allow only two representatives for nearly 12,000 nurses on the College Register. With regard to the General Nursing Council, the College Bill provides that not less than two thirds of the Council shall be elected by postal ballot by nurses on the Register, five-sixths of whom must be nurses on the General Register. The remaining one-third may be representatives of public bodies, training-schools, and representatives of nurses on the Supplementary Register —that is to say, the male nurses and the mental nurses. That Council might consist of forty-five persons, or sixty persons. It would be composed of thirty-six persons, because that is the nearest number to the Bill drawn up by the Central Committee which is divisible by three, and it was when the Central Committee Bill provided for thirty-six that I began to write this paper. I want to take the number, therefore, as thirty-six. Twenty-four out of that thirty-six would have to be nominated and elected by nurses on the General Register. Not less than twenty must be nurses on the General Register. I want you to understand that, because if you do you will understand that half the people on the Council must be nurses on the General Register. No restrictions are made as to the members to be nominated. Members can elect whom they choose. In the Central Committee Bill, out of the General Nursing Council of forty-one only eighteen are direct representatives of the nurses on the General Register. Those nurses may be outnumbered on the Council, and of these representatives eight must be matrons. The public bodies to be represented are to be the same in each case—the Privy Council, the Local Government Board, the British Medical Association, the Medico-Psychological Association, and the nurses' training-schools—with the exception that the College Bill gives representation to the governing bodies of general and Poor-Law hospitals, and the Central Committee merely states four representatives of nurses' training-schools, and further, that the College gives representation to the Board of Education and a Central Committee representative of the medical superintendents of fever hospitals. The representation of the hospitals is, therefore, provided for in each Bill, but the representation under the Central Committee Bill would almost certainly be larger than under the College Bill. One-eighth of the total would be representatives of general and fever hospitals, which is almost certainly larger than they could get under the College Bill. Matrons of hospitals must be elected under the Central Committee Bill, but there is no obligation to nominate matrons at all, if you do not wish, under the College Bill. Whether that is right or not I am not here to say, but you cannot deny that it is a very democratic principle. The Local Government Board is equally represented in this Bill: the same principle for England, Wales, Scotland, and Ireland. In the College Bill representation of Poor-Law Unions is suggested as the best way of representing Poor-Law hospitals. The "other officials" mentioned in this pamphlet can only apply to the representatives of the Board of Education, against whom may be placed the registered medical practitioners to be appointed by the superintendents of fever hospitals. I think you will see, therefore, that the representation of nurses is larger on the College Bill than on the Central Committee Bill.

Removal from Register.

There is another point I would like to mention, because I find it is one on which there is a good deal of misunderstanding. It is often stated that under the College Bill nurses have no means of redress if their names should be removed from the Register. That, I say, is a misunderstanding which is very much abroad. But the nurse has exactly the same right of appeal in the College Bill as she has under the Central Committee Bill. (Hear, hear.) The clauses are almost word for word the same. I would ask all my colleagues here present to think, and to think hard, for themselves. I would very much rather nave an opponent than I would have some flabby person who cannot make up her mind. There is a tremendous need for unity in the profession at the present moment, and this State Registration is so urgently needed that if you could only know how urgent you would wake up. You must wake up. We poor matrons cannot do all the work; you must do some of it yourselves. There is great need for unity, for you to learn, to think, to know how to exercise the vote. Think of the franchise you have got. There were hundreds of nurses in England who might have had the vote but who did not take the trouble to claim it. I want you to wake up and learn to vote on all matters concerning your own profession. The differences between us, I believe, are very small. The differences between these two Bills are very small really; they could be very easily adjusted if people would only make up

**Nurses' Fight for State Registration**—(*continued*).

their minds that they are going to come into agreement, instead of quarrelling about everything which is put forward.

**The Chairman:** The case has now been put, I think you will agree, very adequately for the College Bill, and I shall be glad if any one will get up and speak who is of the opposite opinion. I ask any member here, any member of the audience, who is in doubt on any point, to speak, so that she may be enlightened on it.

### Interesting Debate.

**Miss Rimmer:** I would like to speak from the point of view of the National Union of Trained Nurses. I have listened with great interest to all that has been said this evening, and I have only been regretting all the time that all this energy for State Registration of Nurses was not used to back the Bill that we had got. (Applause.) Then by this time we should have had it. I mean the Bill which the Central Committee for State Registration has been working at for years. The first thing that the nurse members of my Union criticise in your Bill is the word "Limited." We think the College should bear the name without the word "Limited." Why should it not be described as the College of Nursing? Through the Board of Trade the College of Nursing, Limited, first tried to get rid of that name, and then they tried by amalgamating with the Royal British Nursing Association, so as to get possession of their valuable charter, to be known as the Royal College of Nursing. (A Voice: "But we did not amalgamate.") Quite right, the Royal British Nursing Association and the College of Nursing did not amalgamate because the Royal British Nursing Association would not have it in the end, so that fell through. Now they are trying, by an Act of Parliament, to get rid of this word "Limited." I think I may prophesy that the members of the House of Commons will, in all probability, doom this third effort to failure. (Applause.) I think you might very well call this Bill the Bill for the Incorporation of the College of Nursing, Limited, with its Memorandum and Articles of Association. I hope all the nurses who are so eagerly supporting this Bill have carefully studied also the Memorandum and Articles of Association. The College of Nursing, Limited, as an educational body we can welcome; I welcome it heartily, and I would not criticise it if it kept to that. I think we should then all welcome it. There is any amount of educational work to be done for nurses, and members of the National Union of Trained Nurses fail to see why this body, of all the nurses' societies, should have special treatment. By this special treatment an active injustice is done to the individual members of the nursing profession. When the Nursing Council is set up under this proposed Act of Parliament the qualifications of each nurse should be scrutinised, and I claim that in order to do that the two years that the Central Committee for State Registration have asked will be all too short if the nurses are as slow about that part of their business as about others. And during the period of grace a nurse could join without extra examination. Do not be afraid of any other examination, because there will not be any more examinations. (Laughter.) She can be accepted or refused on these qualifications only, and not because she is now on any voluntary Register. (Hear, hear.) This applies—I want to be fair all round—just as much to the members of all the other societies: it applies to members of the Royal British Nurses' Association, to the members of the Irish and Scottish Nursing Associations, and to our National Union of Trained Nurses and other societies. Therefore the members of our Society emphatically protest against this proposal to give preferential treatment to the College of Nursing Register.

### Mr. Lyle, M.P.'s Question.

**Mr. Lyle,** M.P.: May I ask a question, Sir? I do not ask in any spirit of opposition. I am a very humble member of the new Parliament, and I hope to do my utmost there for nurses in every shape and form I can. (Applause.) I may have made a mistake, but I understood Sir Cooper Perry to make a statement which is rather damaging to the small hospitals. I understood him to say that the managers of smaller hospitals were opposing the College Bill because they wished to exploit their nurses. At least, that is how I regarded what he said. I would like, for information only, to know on what evidence he states that that state of affairs exists.

**Sir Cooper Perry:** I have no difficulty in answering. As a matter of fact, I said nothing of the sort. What I think is the fact is, that the State Registration of Nurses will probably make it more difficult for the special hospitals to get probationers. That, I think, is a very real danger, and particularly in the case of children's hospitals. Children's hospitals, in which nurses are trained for three years, will find it difficult to keep up their supply: nurses will not care to go there for three years to be followed by three years' general training. What I think my questioner mistook was this: that there are certain hospitals which do exploit their nurses, not the special ones, not even the small ones—(laughter)—they may be quite big hospitals, even the biggest. Those are the hospitals which exploit their nurses by sending them out to private cases while they are in the course of their training.

### Critics of Supplementary Registers.

**Miss Macdonald:** I want to, perhaps, criticise a clause in this Bill: Clause 4, page 2, dealing with a Supplementary Register. That is a clause which gives great concern to the members of the Association which I have the honour to represent, because they believe it cuts away one of the planks of the State Registrationists, the one-portal system of State Registration. If you are going to establish a Supplementary Register for fever nurses, perhaps one for children's nurses, it may be for health nurses, perhaps for nurses trained in sanatoria, then, under this Bill, you can have, if you like, nurses for the Zoological Gardens. (Laughter and applause.) Those people could all have representation on the Council which is to manage the affairs of this Bill which is supposed to be for fully-qualified trained nurses. I wonder what medical men would think if there were a proposal to put bone-setters on the General Medical Council! Again, supposing Sir Arthur Stanley had a scheme for educating members of the Voluntary Aid Detachments in welfare work and other things, and they were included. I may say I have very great admiration for the work they have done. I admit all that, but in the military hospitals they have been taught nothing of, say, early conditions such as rickets, or the onset of malnutrition—all those conditions of illness which commence so insidiously. You give those people three months' training at the Royal Sanitary Institute, or at the Institute of Public Health, and you institute a special class for them, as you would have power to do under this Bill, and the consent of the Privy Council could easily be got. (Laughter.) You could bring these people under a Health Register. Thus the State would say these half-qualified people are good enough, and so will local authorities say so. In that way

**Nurses' Fight for State Registration—(*continued*).**

this would be putting the seal of the State's approval on people who have quite low qualifications. I have heard that this clause was introduced for the case of children's hospitals. But could not that be done by some good system of reciprocity? In the Royal Infirmary at Edinburgh you will find large wards set aside for this or other special training purpose. Why should not we meet those special hospitals in some way, and get, under the General Nursing Council, a good scheme of reciprocity arranged? (Hear, hear.) The fact is, this particular clause has a very serious effect on the economic position of trained nurses. Behind that clause, as behind the opposition of the last twenty years to State Registration, you have the determination to maintain the reservoir of cheap nursing labour, and a continuance of all the conditions of serfdom which some nurses are working under in hospitals to-day: long hours and the conditions of their work, as well as one or two other points. Sir Cooper Perry spoke of the causes which have delayed State Registration. I think he will admit that was, largely, the opposition encountered from the great training-schools. (Applause.) Over and over again, when we asked for facilities to have our Bill brought before Parliament we were told that it had not the support of the large schools. Then Sir Cooper Perry also talks about the length of the Central Committee's Bill. Is it longer than the Medical Act? (Laughter.) Professor Glaister said there was a period of inaction before the College appeared, and I would remind him of the reason for that. When the war broke out it was decided that controversial Bills should not be introduced, and it was from a spirit of patriotism that the period of inaction ensued (Professor Glaister: it was long before that.) We do not want Supplementary Registers at all, not excepting registers for male and mental nurses.

**The Chairman :** I want to take up at once the point Miss Macdonald gave us, because I think it is the point which has gone home most. It is that of the Supplementary Register. We have put in our Bill that Supplementary Registers *may* be formed. Miss Macdonald has said you have only to get the approval of this Supplementary Register from the Privy Council. But that is not the case; you have got to get the approval of the General Nursing Council which the Bill will set up. Under our Bill two-thirds of the Council have to be nurses on the Register itself. It is in the Bill.

Miss Macdonald : But why have Supplementary Registers at all if you do not wish to keep them?

The Chairman : Because Supplementary Registers may be necessary. If you find it necessary to have Supplementary Registers for male nurses and for mental nurses there may come a time when you will want a Register for other nurses. ("No, never.") But you cannot say there will not be such a time. I have only said there may be a time. I ask you to note this, because it is an important point: no Supplementary Register can be formed under the Bill without the approval of the General Nursing Council, and of that General Nursing Council, under our Bill, two-thirds are nurses elected by the nurses on the State Register. (Applause.) Therefore no Supplementary Register can be formed without the approval of all the nurses themselves who are on the Register. (Applause.)

Miss Rimmer : How about the Provisional Councils? They have time to do that.

The Chairman : It is a misunderstanding of the Bill. A Supplementary Register cannot be passed until the Bill is passed. When the Bill is passed the General Nursing Council comes into effect, and when that comes into effect it comes with our provision that two-thirds are to be nurses elected by the nurses themselves who are on the State Register. Then the nurses will, for the first time, have got control of their own profession.

Miss Rimmer : Not two-thirds of the nurses on the Provisional Council. The Provisional Council can provide a Supplementary Register before you elect the nurses.

The Chairman : No. The Provisional Nursing Council is to draw up rules and regulations, which have to be submitted to the Privy Council for the creation of the General Nursing Council. The Provisional Council can no more make a Supplementary Register than I can.

Miss Macdonald : Will the Council apply to its members when it wishes to form a Supplementary Register? Or will it do it "on its own," so to speak?

The Chairman : Parliament, when it wishes to do anything, does not go back to the electorate to get its permission every time. The nurses on the Council will have been elected by the nurses themselves who are on the Register; therefore if that Council, two-thirds of which have to be nurses, wishes to create a Supplementary Register, it will go to the Privy Council for leave to do so.

Miss Macdonald : You misunderstand me. If this General Nursing Council wishes to establish a Register for first-aid or for fever nurses, will it go to the members of the College and ask their permission to institute this Register, or will the Council do it themselves?

The Chairman : Of course the Council will do it itself; it has been elected for that.

Miss Macdonald : So you will give that Council a blank cheque to institute a Supplementary Register for anything it chooses? It cuts at the root, Sir Arthur, of all the teaching we have had from you.

The Chairman : I think everybody understands the principle. The General Nursing Council that would institute a Supplementary Register if it wishes to do so will consist to the extent of two-thirds, that is, the majority, of nurses who have been elected by the nurses themselves.

(Indistinct talking ensued here.)

**The Chairman : If the nurses will not trust their own elected Council, whom will they trust?**

A Speaker : We have a precedent in the fact that there are general elections, when there is an appeal to the country, as just lately. When there is anything vital to put before the community there is a General Election. If it is thought a Supplementary Register is required would it not be right to approach members on the subject and give them a right to express their opinion on it?

The Chairman : We have it provided in our Bill that two-thirds of the Council are to be nurses. As to the way in which they are to be elected, we have left that for the Privy Council to determine. That is a very real point. I think if the Privy Council were to settle that there was to be an election every year that would be quite right; there would then be fresh blood always coming on to the Council.

Miss Macdonald : So the Council could use this blank cheque at any time they liked? I do not think they should be given that blank cheque.

A Speaker : But if the Council exercises its powers and the electorate uses its powers the new Council can reverse the Register?

The Chairman : Yes.

Professor Glaister : Yes, you could wipe them all out.

**Nurses' Fight for State Registration**—(*continued*).

A Point About Asylum Workers.

Miss Hurlstone: I am asking this question for a matron who is too ill to be here herself. (Indistinctly heard.) It is as to whether members of the National Union of Asylum Workers can be on the Register? Most asylum nurses already belong to that Union, and four of the largest asylums have already been out on strike. (Laughter.)

The Chairman: It is not yet settled up here whether we understand the question.

Miss Hurlstone: They were out for five and a-half days, and then they were reinstated because they had to be given everything they asked for.

The Chairman: What is the point? If they go on like that they will get the vote!

Professor Glaister: Will the lady inform us what associations these are?

Miss Hurlstone: They have got this Union now.

Sir Cooper Perry: The answer is pretty plain, that the Mental Register is formed by those asylum nurses who have passed the examination conducted by the Medico-Psychological Association and approved by the General Nursing Council. None of them are in the College of Nursing at present, because the members only consist of nurses trained in general nursing, and the Bill is not yet passed.

Exploitation of Nurses.

Miss Wheeler: I ask if we could not follow on the lines of the British Medical Association and adopt a certain degree before admitting members. In the medical profession, however clever a man may be, if he is not a medical practitioner he cannot practise. Why should not this Bill be on the same lines? I am a member of the College of Nursing, I am glad to say, but I had some apprehension myself about this clause. It seems to me to be a loophole which might be a big one and undermine the principle we are fighting for. The Member of Parliament asked what evidence we had that nurses in smaller hospitals were being exploited. I myself keep a small nursing home. I sent for two nurses from one institution. There were twelve nurses on the staff, and only one of them possessed a certificate of three years' training, and I know there are very many nurses in the same condition. If every nurse were obliged to be certificated, to possess a certificate of training, I think it would be found that not more than one in twenty would be able to do so showing three years' training. Yet these nurses are going out and taking charge of cases, and some of them have only had three months' training before taking charge of medical and surgical cases. People naturally trust patients to their care, and with a little bluff and some hospital lingo they are taken on, and are receiving the two guineas fee, and so depriving fully-trained nurses of what is their right. Serious cases of illness are left in their hands, cases which they do not understand. When people engage a trained nurse or a nurse from a reputable home they naturally think that the nurse knows everything about the case. She should do. And when these people have an incompetent nurse it arouses doubts in their minds about all nurses, and they do not after that know how far to trust a nurse with a case. It is a matter of real danger. In the provinces we find that very acutely indeed. In London it is not noticed, perhaps because there are so many people. (Applause.)

The Chairman: If I understand rightly, it is exactly in order to remedy that state of things that the College of Nursing has been founded. The whole point and intention of the College of Nurses is to draw the line sharply and distinctly between the woman who is trained and the woman who is untrained.

Miss Wheeler: That is why I am afraid the Supplementary Register may be a loophole. (Applause.) Just as a doctor cannot go on the Medical Register until he has passed certain examinations and spent a certain number of years in training, neither I think should the nurse go on to our Register until she has had the training.

Miss Musson's Doubts.

Miss Musson: I may say I voted consistently against this clause in reference to Supplementary Registers. I need hardly say I make that remark with the consent of the Chairman and of the nurse members of the College of Nursing. As at present constituted, there would not be the slightest chance of any one of them voting for a Supplementary Register other than for mental or male nurses. But those who advised on the drawing up of the Bill thought that if we did not put in a clause such as this Parliament would probably insist on introducing other clauses which we should like still less. And I, as a fully-trained nurse, think that when the next election is on nurses will take great care to elect on to the Council only those who are trained to stand up for the Registration of Nurses.

Miss Macdonald: I would like to ask what Parliament would do which would be worse than introducing Supplementary Registration?

Mrs. Shirley: I think we should be better without a Bill at all than one which allowed Supplementary Registers to be formed, so if that clause is put in to get the Bill through it should be defeated.

Miss Wheeler: What I am anxious for is that every nurse shall be labelled aright.

The Chairman: Surely that is what a Supplementary Register would do; it would label a nurse a "fever nurse" or a "children's nurse," as the case may be.

Miss Macdonald: But if nurses put "Registered Nurse" after their name, as they would have the right to do, would any member of the public ask a nurse calling herself a Registered Nurse, "Are you a fever nurse, or a general nurse, or a mental nurse?" They do not even ask for certificates of nurses now; they look at the uniform.

Removal from the Register.

Miss Breay: There is the question about a nurse being removed from the Register. There is some misapprehension about that. It was not in the Bill at first. It was at the instance of Mrs. Bedford Fenwick, whose name is one which must be always mentioned in connection with State Registration, that that clause was put in. But it is not in the Memorandum. You can turn anybody off the College of Nursing that you please, and she has no appeal. It is put into the Nurses' Registration Bill that any nurse who belongs to the College of Nursing can be turned off, and there is no appeal at all.

Professor Glaister: It says the Memorandum and Articles of Association shall only be in force so far as they are in consistence with the Bill, and there must be an appeal.

College Register v. State Register.

Miss Breay (in further comment): I am talking about the present, not about the future. The other thing is you tell people whom you wish to join the College that they should join it because the Council of the College of Nurses has drafted a Nurses' Registration Bill which provides that the Register already formed by the College of Nurses shall be a first Register under the Act. If

**Nurses' Fight for State Registration**—(*continued*).
therefore you are on the College Register you will automatically and without further fee be placed upon the State Register when the Nurses' Registration Bill is passed. I ask what ground there is for that statement. How can you pledge Parliament to that?

THE CHAIRMAN: We do not pledge Parliament to it. We tell people we are bringing forward this College Bill, and if the College Registration Bill is passed members shall be on.

MISS MACDONALD: Because the College of Nursing has drafted a Registration Bill. If therefore you are on the College Register—not if the College Bill is passed—if you are on the College Register, you will automatically and without further fee be placed on the State Register when this Bill is passed.

THE CHAIRMAN: We will not quibble about words. I think the opening sentence makes the thing perfectly clear. As regards people on the Register of the College, I do not think any members on the Register need have any fear as to the future. If they are qualified to be on the College Register they can be perfectly certain they will be qualified to be on the State Register. They will be qualified because our Register has been much more strictly formed, I should think, than the State Register is likely to be. I know, because I have tried to get nurses on it and I have invariably been refused. And the other part of the question which says they will go on the Register without fee I can answer for. Having paid one guinea fee already the College itself will look to it that if there is a further fee to be paid it will be paid by the College. (Applause.)

GROWING ENDOWMENT FUND.

MISS MACDONALD: Then what will the College work on if its membership subscriptions are to go away in this form?

THE CHAIRMAN: It is not giving away a secret when I say that the College endowment fund is getting on very nicely. Apart from what we have received in other ways we had the pleasure of reporting the receipt to-day of a cheque for £15,000—(applause)—and I am confidently expecting to report at the next meeting of the Council a sum which I know has already amounted to £12,000. So there need not be any uneasiness as to the College funds.

MISS MACDONALD: If you are going to have a Council like this representative of the employers of the nurses and officials financed by large sums of money—(hear, hear)—you will be handing over the profession as a body to them.

THE CHAIRMAN: At all events, 12,000 members of the profession are taking very kindly to it. (Laughter and applause.)

THE RIGHT OF APPEAL ON EXPULSION.

SIR HENRY BURDETT, K.C.B.: A lady behind (Miss Breay) has asked a question, and I should like Sir Cooper Perry to answer it; he has not done so yet. She is under the impression that the constitution of the College as it stands at present provides for the ejection of members on the College Register without any right of appeal whatever. I want to know whether that is correct. I think it is not correct, and we shall be glad to hear.

SIR COOPER PERRY: I think that, technically, what the lady says is correct, but she has forgotten to add that in order to get rid of a member there have to be two-thirds of the whole Council satisfied that she is properly got rid of. ("That does not matter.") It is a well known fact that no club can exclude a member without giving that member an opportunity of being heard, because if the case goes into the Courts the Courts may restrain the Club or the Council from getting rid of the member. That is a security which is inherent in English law. It is the greatest security we can have; it is the same security that the nurse has under the Bill. The reason it has to be put in the Bill is that the Bill erects a Council and gives it powers, and there must be some clause by which these powers are limited by reference to the Courts. That clause does not exist in the Bill under which we doctors are registered, and we are liable to be removed for "infamous conduct in a professional respect," whatever that may mean, by the General Medical Council without appeal, so that we poor things are liable to a sad injustice. This is a College of Nursing we are speaking of, therefore the nurse has that appeal under the Bill and an appeal in a Court of Law if she thinks she is aggrieved.

A SPEAKER: If we had one badge for Registered Nurses only there would be something in that.

THE CHAIRMAN: Ah, that is getting on *some* way. I sincerely hope that we shall have that. I say "we," although I am not a nurse.

MISS BREAY: Is the College prepared to frank the expense of a nurse who would appeal?

THE CHAIRMAN: It is the first time I have heard of an association suggesting paying money for anybody to appeal against it. The College is prepared to go far, but I do not know whether it will go all that way.

DISTRUSTFUL NURSES.

MISS COX-DAVIES: The common sense point of view appears to me to be that the ladies who have spoken to-night do not trust their fellow-nurses very greatly. (Applause.) I am still a nurse though I am a matron, and I have lived among nurses for more years than I care to say in a meeting such as this, but I do really trust my fellow-nurses absolutely, and when two-thirds of them on the Council are elected by nurses themselves, I am prepared to trust them to see that things that are injurious for the profession are not done. (Applause.)

CHAIRMAN'S PLEA FOR UNITY.

THE CHAIRMAN: Well now, Ladies and Gentlemen, time is getting on, and I think we have had a most interesting discussion this evening. And I think really what has come out of this discussion is exactly what has been my experience since I came, innocently, into this question at all. (Laughter.) It is that as a matter of fact between the whole of the contending parties there is very little difference indeed, and I believe that if half-a-dozen sensible people could sit at a round table we should have a simple Bill to-morrow. That would be possible if we were to try and forget our trials and difficulties of the past. The principles of both Bills are essentially the same. We want a State Registration of properly trained nurses, and that is the foundation of the whole thing.

THE V.A.D. SCHOLARSHIP SCHEME.

I have only one word to say in conclusion, and in no connection with this Bill. It is simply because I happen to have the good fortune to be speaking for a large number of nurses. I have seen it in print, and I have heard it stated on more than one occasion—I have seen it in print on one occasion, or at all events it was implied in print—that the scheme of the Red Cross and Order of St. John of Jerusalem for V.A.D. scholarships is in some way a move on the part of the College of Nursing for introducing V.A.D.s into the College of Nursing. I wish to say in plain language that anybody who says or implies that is telling an absolute lie. They have got absolutely nothing to do with one another. The V.A.D. scholarship scheme was brought forward by the Red Cross and Order

**Nurses' Fight for State Registration**—(*concluded*).
of St. John of Jerusalem as a help in the middle of their training to those girls who came forward in the war, and it wanted to mark, as I think the country has desired to do, what they did then. It has no more connection with the College of Nursing than with the man in the [illegible]. One lady has gone further than she perhaps in[illegible]ded to do, and says that some part of the Nation's Fund for Nurses is being used for this College scheme. That is absolutely, literally, untrue; there is not a single word of truth in it at all. If you hear anybody mixing up the V.A.D. Scholarship Scheme with the College of Nursing you may tell them that they know absolutely nothing about it, and that they had better hold their tongue.

ALL IMPORTANT TO NURSES.

**I think I now need only to thank you, as Chairman, for your great kindness in coming in such numbers this evening. At all events, nobody can say that the interest of the nurses in their own profession is not thoroughly awakened, and I am certain you realise how necessary it is to be united and to work for yourselves. I should like to echo the words of Miss Musson and to remind you that Matrons, or even those awful people the employers of nurses, cannot poss bly do everything, and that the work must be done by the nurses themselves. (Hear, hear.) We are looking after certain sides of the question such as the money, and if you will give us the necessary driving force by joining the College, if you will enable us to go to Parliament not with 12,000 members, but with 20,000, 25,000, or even 30,000, you can be certain that you will have a State Registration Bill, and that it will be one which, on whichever side you are, you will all heartily agree and support. (Applause.)**

CONFIDENCE IN THE COLLEGE.

MISS ALSOP: I ask members to pass a very hearty vote of thanks to Sir Arthur Stanley and the other speakers for the patience they have had in talking to us about the College of Nursing. I think the fact of 12,000 nurses belonging to it, all sensible women above the age of twenty-one, and therefore persons who can be trusted with the lives of their patients, who know that they are electing their own Council, shows that we have got the greatest confidence in the College of Nursing, as well as all that it has done, and we wish it every success.

The resolution was carried by acclamation.

As the meeting was dispersing Miss Montgomery invited those present to visit the new premises in Henrietta Street and to join the College Centre, the annual subscription of which is 5s.

